# Pull-back myotomy to prevent mucosal injury during peroral endoscopic myotomy for jackhammer esophagus

**DOI:** 10.1055/a-2301-8035

**Published:** 2024-04-24

**Authors:** Jun Nakamura, Takuto Hikichi, Minami Hashimoto, Tsunetaka Kato, Takumi Yanagita, Tadayuki Takagi, Hiromasa Ohira

**Affiliations:** 1 215686 Department of Endoscopy, Fukushima Medical University Hospital, Fukushima, Japan; 2 183174 Department of Gastroenterology, Fukushima Medical University School of Medicine, Fukushima, Japan

Mucosal injury is a notable perioperative complication of peroral endoscopic myotomy (POEM), occurring in 1.6%–25.8% of procedures 1 2 3 . Esophageal perforation caused by mucosal injury can lead to leakage of contents into the mediastinum, potentially resulting in mediastinitis 4 . Jackhammer esophagus is a hypercontractile esophageal motility disorder diagnosed using high-resolution manometry (HRM), necessitating extended myotomy in POEM 5 . Additionally, heightened caution is warranted when the length of the submucosal tunnel exceeds 13 cm, as it is associated with an elevated risk of mucosal injury 2 . Therefore, the POEM for jackhammer esophagus should be approached cautiously, considering the potential risk of mucosal injury.

Herein, we report on a 74-year-old man who underwent POEM of jackhammer esophagus. Endoscopic examination showed spastic contractions in the esophageal body impeded the passage of the scope. HRM showed hypercontractility of the esophageal body ( Fig. 1 ). We performed the POEM using a Triangle Tip Knife J (Olympus, Tokyo, Japan). Hypercontraction was observed endoscopically during submucosal tunnel creation and myotomy. Initially, we started a conventional myotomy, making an incision from the muscle side to the tunnel side. However, concerns arose regarding mucosal injury due to contact between the knife and the mucosa during hypercontraction ( Fig. 2 **a** ). Therefore, we converted to an alternative procedure termed “pull-back myotomy”, moving the Triangle Tip Knife J from the tunnel to the muscle layer ( Fig. 2 **b** – **d** , Video 1 ). This approach effectively prevented knife contact with the mucosa, even during hypercontraction. Consequently, a 19-cm myotomy was completed without causing mucosal injury. Four months after POEM, hypercontraction had disappeared, and the patient’s symptoms were improved ( Fig. 3 ).

**Fig. 1:**
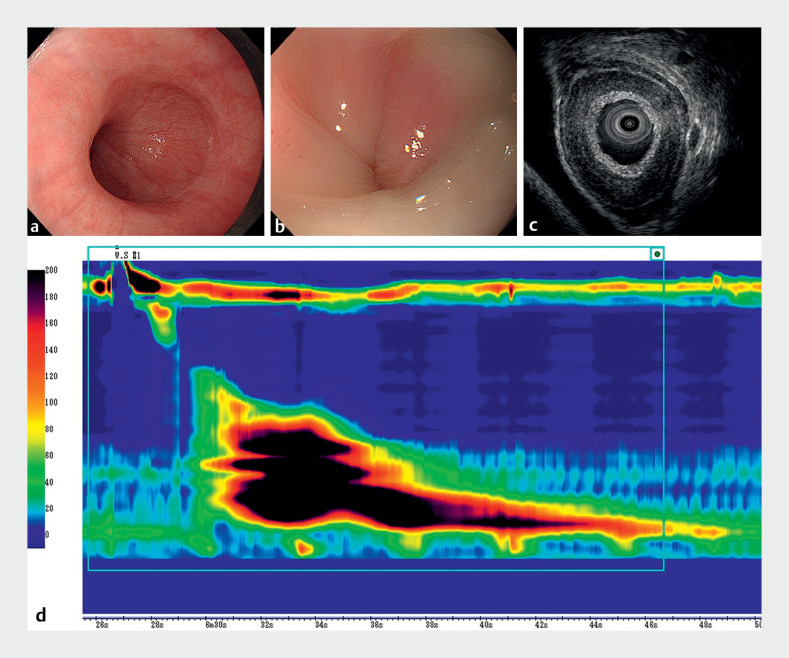
Examinations before peroral endoscopic myotomy. **a** Normal esophageal peristalsis was not observed during endoscopy. **b** Abnormally strong contractions were observed. **c** Endoscopic ultrasonography showed 3 mm thickening of the inner circular muscle of the esophageal body. **d** High-resolution manometry showed hypercontractility, with the highest distal contractile integral reaching 22,488 mmHg·cm·s; over 40% of swallows had distal contractile integral values exceeding 8,000 mmHg·s·cm.

**Fig. 2:**
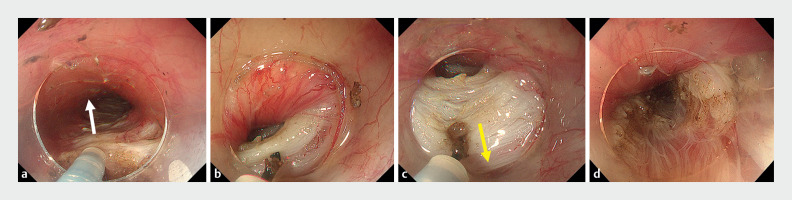
Endoscopic findings during peroral endoscopic myotomy. **a** After creating a submucosal tunnel, conventional myotomy was initiated using the Triangle Tip Knife J from the muscle layer side to the submucosal tunnel side (white arrow). **b** Concerns about mucosal injury arose due to the proximity of the mucosa and muscle during strong contractions. **c** During the pull-back myotomy, the Triangle Tip Knife J was operated from the submucosal tunnel to the muscle layer (yellow arrow), thereby preventing mucosal injury. **d** Myotomy was completed without mucosal injury.

**Fig. 3:**
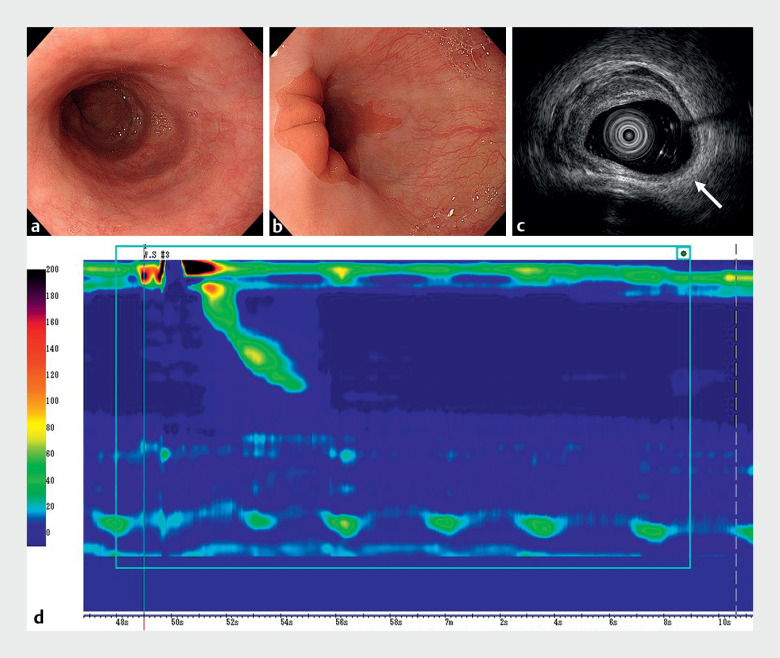
Examinations after peroral endoscopic myotomy. **a** Endoscopy showed no abnormal contraction of the esophagus. **b** There were no findings of gastroesophageal reflux disease at the esophagogastric junction. **c** Endoscopic ultrasonography showed that the thickening of the muscle layer had disappeared in the area where the myotomy was performed (white arrow). **d** Abnormal contractions also disappeared on high-resolution manometry.

Pull-back myotomy to prevent mucosal injury during peroral endoscopic myotomy for jackhammer esophagus.Video 1

To the best of knowledge, there are no reports detailing specific myotomy techniques designed to prevent mucosal injury, such as the pull-back myotomy. However, large-scale studies are needed to determine the efficacy of this procedure.

Endoscopy_UCTN_Code_TTT_1AO_2AP

## Notes

Endoscopy E-Videos https://eref.thieme.de/e-videos
*E-Videos* is an open access online section of the journal *Endoscopy* , reporting on interesting cases and new techniques in gastroenterological endoscopy. All papers include a high-quality video and are published with a Creative Commons CC-BY license. Endoscopy E-Videos qualify for HINARI discounts and waivers and eligibility is automatically checked during the submission process. We grant 100% waivers to articles whose corresponding authors are based in Group A countries and 50% waivers to those who are based in Group B countries as classified by Research4Life (see: https://www.research4life.org/access/eligibility/ ). This section has its own submission website at https://mc.manuscriptcentral.com/e-videos .
